# Supplementary material for: Multi-phosphorylation reaction and clustering tune Pom1 gradient mid-cell levels according to cell size
Source: eLife. 2019 May 3;8:e45983. doi: 10.7554/eLife.45983 (PMC6555594; doi:10.7554/eLife.45983)
Supplement: Supplementary file 1. [file elife-45983-supp1.docx]

**Supplementary File 1: *S. pombe* strains used in this study.**

| **Number** | **Genotype** | **Source** |
| --- | --- | --- |
| **Figure 1-2** | | |
| YSM3262 | h- pom1-mEos3.2-kanMX ade6-M216 leu1-32 ura4-D18 | This study. |
| YSM3263 | h+ pom1^KD^-mEos3.2-kanMX leu1-32 ura4-D18 | This study. |
| YSM3264 | h+ pom1^3A(2,4,5)^-mEos3.2-kanMx ade6-M216 leu1-32 ura4-D18 | This study. |
| **Figure 3** | | |
| YSM3265 | h- ura4-D18::*ppom1*-pom1_(305-510)_-GFP::ura4+ ade6-M210 leu1-32 | This study. |
| YSM3266 | h- ura4-D18::*ppom1*-pom1_(305-490)_-GFP::ura4+ ade6-M210 leu1-32 | This study. |
| YSM3267 | h- ura4-D18::*ppom1*-pom1_(305-473)_-GFP::ura4+ ade6-M210 leu1-32 | This study. |
| YSM3268 | h- ura4-D18::*ppom1*-pom1_(419-510)_-GFP::ura4+ ade6-M210 leu1-32 | This study. |
| YSM3269 | h- ura4-D18::*ppom1*-pom1_(468-510)_-GFP::ura4+ ade6-M210 leu1-32 | This study. |
| YSM3270 | h- ura4-D18::*ppom1*-pom1_(468-510)_^I494N^-GFP::ura4+ ade6-M210 leu1-32 | This study. |
| YSM3271 | h- ura4-D18::*ppom1*-pom1_(305-510)_^I494N^-GFP::ura4+ ade6-M210 leu1-32 | This study. |
| YSM3272 | h- ura4-D18::*ppom1*-pom1_(305-510)_^7ALA(MB1*)^-GFP::ura4+ ade6-M210 leu1-32 | This study. |
| YSM3273 | h- ura4-D18:: *ppom1*- _(305-510)_^I494N-MB1*^-GFP::ura4+ ade6-M210 leu1-32 | This study. |
| YSM3274 | h- pom1-GFP-kanMX ade6-M210 leu1-32 ura4-D18 | This study. |
| YSM3275 | h- pom1^I494N^ -GFP-kanMX ade6-M210 leu1-32 ura4-D18 | This study. |
| YSM3276 | h- pom1^7ALA(MB1*)^ -GFP-kanMX ade6-M210 leu1-32 ura4-D18 | This study. |
| YSM3277 | h- pom1^I494N-MB1*^ -GFP-kanMX ade6-M210 leu1-32 ura4-D18 | This study. |
| YSM3278 | h- pom1^I494N-MB1*-5PxxP*^ -GFP-kanMX ade6-M210 leu1-32 ura4-D18 | This study. |
| **Figure S3** | | |
| YSM3279 | pom1Δ::kanMX ura4-D18::pom1_(305-510)_-GFP::ura4+ ade6-M210 leu1-32 | This study. |
| YSM3280 | pom1Δ::kanMX ura4-D18::pom1_(305-490)_-GFP::ura4+ ade6-M210 leu1-32 | This study. |
| YSM3281 | pom1Δ::kanMX ura4-D18::pom1_(305-473)_-GFP::ura4+ ade6-M210 leu1-32 | This study. |
| YSM3282 | pom1Δ::kanMX ura4-D18::pom1_(419-510)_-GFP::ura4+ ade6-M210 leu1-32 | This study. |
| YSM3283 | pom1Δ::kanMX ura4-D18::pom1_(468-510)_-GFP::ura4+ ade6-M210 leu1-32 | This study. |
| YSM3284 | pom1Δ::kanMX ura4-D18::pom1_(468-510)_^I494N^-GFP::ura4+ ade6-M210 leu1-32 | This study. |
| YSM3285 | pom1Δ::kanMX ura4-D18::pom1_(305-510)_^7ALA(MB1*)^-GFP::ura4+ ade6-M210 leu1-32 | This study. |
| **Figure 4** | | |
| YSM1912 | h- pom1-GFP-kanMX ade6-M216 leu1-32 ura4-D18 | (Hachet et al., 2011) |
| YSM3287 | h+ pom1^1A(5)^-GFP-kanMX ade6-M216 leu1-32 ura4-D18 | This study. |
| YSM3288 | h+ pom1^2A(4,5)^-GFP-kanMX ade6-M216 leu1-32 ura4-D18 | This study. |
| YSM3289 | h+ pom1^3A(2,4,5)^-GFP-kanMX ade6-M216 leu1-32 ura4-D18 | This study. |
| YSM3290 | h+ pom1^5A(1,2,3,4,5)^-GFP-kanMX ade6-M216 leu1-32 ura4-D18 | This study. |
| YSM2271 | h+ pom1^6A(1,2,3,4,5,6)^-GFP-kanMX ade6-M216 leu1-32 ura4-D18 | (Hachet et al., 2011) |
| YSM1329 | h- pom1^KD^-GFP-kanMX ade6-M216 leu1-32 ura4-D18 | (Hachet et al., 2011) |
| YSM3291 | tea4Δ::hphMX pom1-GFP-kanMX ade6-M216 leu1-32 ura4-D18 | This study. |
| YSM3292 | tea4Δ::hphMX pom1^1A(5)^-GFP-kanMX ade6-M216 leu1-32 ura4-D18 | This study. |
| YSM3293 | tea4Δ::hphMX pom1^2A(4,5)^-GFP-kanMX ade6-M216 leu1-32 ura4-D18 | This study. |
| YSM3294 | tea4Δ::hphMX pom1^3A(2,4,5)^-GFP-kanMX ade6-M216 leu1-32 ura4-D18 | This study. |
| YSM3295 | tea4Δ::hphMX pom1^5A(1,2,3,4,5)^-GFP-kanMX ade6-M216 leu1-32 ura4-D18 | This study. |
| YSM3296 | tea4Δ::hphMX pom1^6A(1,2,3,4,5,6)^-GFP-kanMX ade6-M216 leu1-32 ura4-D18 | This study. |
| YSM1855 | tea4Δ::kanMX pom1^KD^-GFP-kanMX ade6-M210 leu1-32 ura4-D18 | (Hachet et al., 2011) |
| YSM3274 | h- pom1-GFP-kanMX ade6-M210 leu1-32 ura4-D18 | This study. |
| YSM3276 | h- pom1^7ALA(MB1*)^-GFP-kanMX ade6-M210 leu1-32 ura4-D18 | This study. |
| Ysm3286 | h- pom1^7ALA(MB1*)-1(A5)^-GFP-kanMX ade6-M210 leu1-32 ura4-D18 | This study. |
| **Figure 5** | | |
| YSM1912 | h- pom1-GFP-kanMX ade6-M216 leu1-32 ura4-D18 | (Hachet et al., 2011) |
| YSM3289 | h+ pom1^3A(2,4,5)^-GFP-kanMX ade6-M216 leu1-32 ura4-D18 | This study. |
| YSM1413 | h- cdr2-GFP-ura4+ ade6-M216 leu1-32 ura4-D18 | (Bhatia et al., 2014) |
| YMM773 | h- ura4-D18::ppom1-sfGFP-tCYC1::ura4+ ade+ leu+ | This study. |
| YSM165 | h- tea4Δ::kanMX pom1-GFP-kanMX ade6-M210 leu1-32 ura4-D18 | (Martin et al., 2005) |
| **Figure 6** | | |
| YSM1457 | pom1-GFP-kanMX cdr2-tdTomato-natMX | This study. |
