# Supplementary material for: Multi-phosphorylation reaction and clustering tune Pom1 gradient mid-cell levels according to cell size
Source: eLife. 2019 May 3;8:e45983. doi: 10.7554/eLife.45983 (PMC6555594; doi:10.7554/eLife.45983)
Supplement: Supplementary file 2. [file elife-45983-supp2.docx]

**Supplementary File 2: Plasmid used in this study.**

| Number | Description | Purpose |
| --- | --- | --- |
| pSM2114 | pAV0133-*ppom1*-pom1_(305-510)_-GFP | *ura4* integration |
| pSM2115 | pAV0133-*ppom1*-pom1_(305-490)_-GFP | *ura4* integration |
| pSM2116 | pAV0133-*ppom1*-pom1_(305-510)_^7ALA(MB1*)^-GFP | *ura4* integration |
| pSM2117 | pAV0133-*ppom1*-pom1_(305-473)_-GFP | *ura4* integration |
| pSM2118 | pAV0133-*ppom1*-pom1_(419-510)_-GFP | *ura4* integration |
| pSM1848 | pAV0133-*ppom1*-pom1_(419-510)_-GFP | *ura4* integration |
| pSM1850 | pAV0133-*ppom1*-pom1_(419-510)_ ^I494N^-GFP | *ura4* integration |
| pSM2132 | pAV0133-*ppom1*-pom1_(305-510)_ ^I494N^-GFP | *ura4* integration |
| pSM2133 | pAV0133-*ppom1*-pom1_(305-510)_ ^MB1*-I494N^-GFP | *ura4* integration |
| pSM2142 | pFA6A-*ppom1-*pom1ORF-GFP-kanMX- *pom1 3`UTR* | target endogenous locus |
| pSM2146 | pFA6A-*ppom1-*pom1 ^I494N^-GFP-kanMX- *pom1 3`UTR* | target endogenous locus |
| pSM2237 | pFA6A-*ppom1-*pom1 ^MB1*^-GFP-kanMX- *pom1 3`UTR* | target endogenous locus |
| pSM2238 | pFA6A-*ppom1-*pom1 ^MB1*-I494N^-GFP-kanMX- *pom1 3`UTR* | target endogenous locus |
| pSM2264 | pFA6A-*ppom1-*pom1 ^MB1*-1A(5)^-GFP-kanMX- *pom1 3`UTR* | target endogenous locus |
| pSM2328 | pFA6A-*ppom1-*pom1 ^MB1*-I494N-5PxxP*^-GFP-kanMX-*3`UTR* | target endogenous locus |
| pSM738 | pREP41-pom1-GFP | phosphosite mutagenesis |
| pSM1502 | pREP41-pom1^1A(5)^ | phosphosite mutagenesis |
| pSM1576 | pREP41-pom1^2A(2,5)^ | phosphosite mutagenesis |
| pSM1527 | pREP41-pom1^3A(2,4,5)^ | phosphosite mutagenesis |
| pSM1866 | pREP41-pom1^5A(1,2,3,4,5)^ | phosphosite mutagenesis |
| pSM1731 | pFA6A- pom1_(911-1087)_-GFP-kanMX-*pom1 3`UTR* | C-terminal GFP tagging |
