# Supplementary material for: Multi-phosphorylation reaction and clustering tune Pom1 gradient mid-cell levels according to cell size
Source: eLife. 2019 May 3;8:e45983. doi: 10.7554/eLife.45983 (PMC6555594; doi:10.7554/eLife.45983)
Supplement: Supplementary file 3. — Bold residues indicate changes from the wildtype sequence. [file elife-45983-supp3.docx]

**Supplementary File 3: Primers used for mutagenesis.** Bold residues indicate changes from the wildtype sequence.

| Mutation | 5’-3’ sequence |
| --- | --- |
| Pom1^MB1*^ fragment for | GGTAGATCTTC**AgcAGcTGcCgcTgcTgcCgc**ATTGAGTTTTTCGAGAAGTTCTTCTC |
| Pom1^MB1*^ fragment rev | GCTCTCGAAGTGCAGAATCACTATTACCGTTAG |
| Pom1^I494N^ for | AGCCGGATGCAGCAATTT**AAT**AACTGGTTCAAACCATCC |
| Pom1^I494N^ rev | GGATGGTTTGAACCAGTT**ATT**AAATTGCTGCATCCGGCT |
| MB1*3ALA for | acaaagaaggtagatcttca**gcAGcTGcC**ttttttagcagattgagttt |
| MB1*3ALA rev | aaactcaatctgctaaaaaa**GgCAgCTgc**tgaagatctaccttctttgt |
| MB1*5ALA for | GTAGATCTTCAGCAGCTGCC**gcTgcT**AGCAGATTGAGTTTTTCGAG |
| MB1*5ALA rev | CTCGAAAAACTCAATCTGCT**AgcAgc**GGCAGCTGCTGAAGATCTAC |
| MB1*7ALA (MB1*) for | CTTCAGCAGCTGCCGCTGCT**gcCgcA**TTGAGTTTTTCGAGAAGTTC |
| MB1*7ALA (MB1*) rev | GAACTTCTCGAAAAACTCAA**TgcGgc**AGCAGCGGCAGCTGCTGAAG |
| PxxP site4 for | CCCGAGGAAATT**G**CTTCAGTT**G**CTCCACTACCTTC |
| PxxP site4 rev | GAAGGTAGTGGAGCAACTGAAGCAATTTCCTCGGG |
| PxxP site5 for | CCGCGTCCTTTA**G**CGAATTTG**G**CAATGGAATACAATG |
| PxxP site5 rev | CATTGTATTCCATTGCCAAATTCGCTAAAGGACGCGG |
| Pom1^SS427-8AA^ site1 for | CAACAAAGAAGGTAGA**G**CT**G**CAAGAGGTGGCTTTTTTAG |
| Pom1^SS427-8AA^ site1 rev | CTAAAAAAGCCACCTCTTGCAGCTCTACCTTCTTTGTTG |
| Pom1^SFS437-9AFA^ site2 for | GCTTTTTTAGCAGATTG**GC**TTTT**G**CGAGAAGTTCTTCTC |
| Pom1^SFS437-9AFA^ site2 rev | GAGAAGAACTTCTCGCAAAAGCCAATCTGCTAAAAAAGC |
| Pom1^TPT482-4APA^ site4 for | CCGGAATGGAAAGAAA**G**CTCCT**G**CGAGAACGAAAAGCC |
| Pom1^TPT482-4APA^ site4 rev | GGCTTTTCGTTCTCGCAGGAGCTTTCTTTCCATTCCGG |
| Pom1^S488A^ site5 f | CCTACGAGAACGAAA**GC**CCGGATGCAGCAATTT |
| Pom1^S488A^ site5 r | AAATTGCTGCATCCGGGCTTTCGTTCTCGTAGG |
