# Supplementary material for: Multi-phosphorylation reaction and clustering tune Pom1 gradient mid-cell levels according to cell size
Source: eLife. 2019 May 3;8:e45983. doi: 10.7554/eLife.45983 (PMC6555594; doi:10.7554/eLife.45983)
Supplement: Supplementary file 4. [file elife-45983-supp4.docx]

**Supplementary File 4: Global fitting parameters and outputs for diffusion and dissociation coefficients.**

|  |  | **Dissociation** | | | | | | | **Diffusion** | | |
| --- | --- | --- | --- | --- | --- | --- | --- | --- | --- | --- | --- |
|  |  |  | ***Input*** | | | ***Output*** | | | ***Mean Diff. Coef.*** | | |
| **Cond.** | ***Pop.*** | $\tau_{\mathrm{TL}}$  *[*s*]* | ***Fixed Par.*** | ***Boundary***  ***Cond.*** | | $k_{\mathrm{bleach}}$  *[*s^-1^*]* | $k_{\mathrm{off}}$  *[*s^-1^*]* | $t_{\mathrm{off}}$  *[*s*]* | ***_D_ Bound.***  ***[***um^2^/s***]*** | ***Pole***  ***10^x^***  ***[***um^2^/s***]*** | ***@3um from pole***  ***10^x^ [***um^2^/s***]*** |
| WT | Fast | 0.2 | $t_{\mathrm{int}}$  $\tau_{\mathrm{TL}}$ | $t_{bleach}>0$ | $t_{\mathrm{eff}}<$0.56 | 6.5±2.0 | 0.90±0.69 | 1.1±0.7 | $D_{\mathrm{coef}}>$10^-1^ | -0.91±0.05 | -0.58±0.05 |
|  |  | 0.120 |  |  | $t_{\mathrm{eff}}<$1.29 |  |  |  |  |  |  |
|  |  | 0.220 |  |  | $t_{\mathrm{eff}}<$1.78 |  |  |  |  |  |  |
|  | Slow | 0.2 | $t_{\mathrm{int}}$  $\tau_{\mathrm{TL}}$  $t_{bleach}$ |  | 0.56 $<t_{\mathrm{eff}}<$  1.83 |  | 0.32±0.27 | 3.1±0.8 | 10^-2^ < $D_{\mathrm{coef}}<$10^-1^ | -1.68±0.02 | -1.60±0.02 |
|  |  | 0.120 |  |  | 1.29 $<t_{\mathrm{eff}}<$  3.86 |  |  |  |  |  |  |
|  |  | 0.220 |  |  | 1.78 $<t_{\mathrm{eff}}<$  7.50 |  |  |  |  |  |  |
| KD | Fast | 0.2 | $t_{\mathrm{int}}$  $\tau_{\mathrm{TL}}$ |  | $t_{\mathrm{eff}}<$0.63 | 6.0±1.9 | 0.53±0.21 | 1.9±0.4 | $D_{\mathrm{coef}}>$10^-1.4^ | - | -1.19±0.04 |
|  |  | 0.120 |  |  | $t_{\mathrm{eff}}<$1.39 |  |  |  |  |  |  |
|  |  | 0.220 |  |  | $t_{\mathrm{eff}}<$3.5 |  |  |  |  |  |  |
|  | Slow | 0.2 | $t_{\mathrm{int}}$  $\tau_{\mathrm{TL}}$  $t_{bleach}$ |  | 0.63 $<t_{\mathrm{eff}}<$  1.79 |  | 0.19±0.20 | 5.2±1.1 | 10^-2^ $<D_{\mathrm{coef}}<$10^-1.4^ | - | -1.77±0.01 |
|  |  | 0.120 |  |  | 1.39 $<t_{\mathrm{eff}}<$  4.18 |  |  |  |  |  |  |
|  |  | 0.220 |  |  | 3.5 $<t_{\mathrm{eff}}<$  8.11 |  |  |  |  |  |  |
| 3A | Fast | 0.2 | $t_{\mathrm{int}}$  $\tau_{\mathrm{TL}}$ |  | $t_{\mathrm{eff}}<$0.3 | 5.5±0.3 | 0.60±0.04 | 1.7±0.1 | $D_{\mathrm{coef}}>$ 10^-1^ | - | -0.6±0.31 |
|  |  | 0.120 |  |  | $t_{\mathrm{eff}}<$0.84 |  |  |  |  |  |  |
|  |  | 0.220 |  |  | $t_{\mathrm{eff}}<$1.1 |  |  |  |  |  |  |
|  | Slow | 0.2 | - |  | 0.1 $<t_{\mathrm{eff}}$ |  | - | - | 10^-2^ $<D_{\mathrm{coef}}<$10^-1^ | - | -1.70±0.05 |
|  |  | 0.120 |  |  | 0.84 $<t_{\mathrm{eff}}$ |  |  |  |  |  |  |
|  |  | 0.220 |  |  | 1.1 $<t_{\mathrm{eff}}$ |  |  |  |  |  |  |
